# Clinical and paraclinical characteristics of optic neuritis in the context of the McDonald criteria 2017

**DOI:** 10.1038/s41598-024-57199-4

**Published:** 2024-03-27

**Authors:** Konstantin F. Jendretzky, Anna Bajor, Lisa-Marie Lezius, Martin W. Hümmert, Franz Felix Konen, Gerrit M. Grosse, Philipp Schwenkenbecher, Kurt-Wolfram Sühs, Corinna Trebst, Carsten Framme, Mike P. Wattjes, Sven G. Meuth, Stefan Gingele, Thomas Skripuletz

**Affiliations:** 1 Department of Neurology, Hannover Medical School, Carl-Neuberg-Str. 1, 30625 Hannover, Germany; 2 Department of Ophthalmology, Hannover Medical School, Hannover, Germany; 3 Institute for Diagnostic and Interventional Neuroradiology, Hannover Medical School, Hannover, Germany; 4 Department of Neurology, Medical Faculty, Heinrich Heine University Düsseldorf, Düsseldorf, Germany

**Keywords:** Optic neuritis, Multiple sclerosis, Clinically isolated syndrome, McDonald Criteria, Diagnostic criteria, Multiple sclerosis, Neurology, Autoimmunity

## Abstract

Optic neuritis is often an initial symptom in multiple sclerosis (MS) or clinically isolated syndrome (CIS), yet comprehensive studies using the 2017 McDonald criteria for MS are scarce. Patient records from our academic centre (2010–2018) were reviewed. Using the 2017 McDonald criteria, three groups were formed: MS optic neuritis (optic neuritis with confirmed MS), CIS optic neuritis (optic neuritis without confirmed MS) and suspected optic neuritis (sON). We compared clinical and paraclinical findings among the groups to identify predictors for CIS- or MS-optic neuritis. The study included 129 MS, 108 CIS, and 44 sON cases. The combination of visual impairment, dyschromatopsia, and retrobulbar pain was observed in 47% of MS patients, 42% of CIS patients, and 30% of sON patients. Dyschromatopsia was the strongest indicator of MS or CIS diagnosis in the backward regression model. 56% of MS patients had relative afferent pupillary defect, 61% optic nerve anomalies within magnetic resonance imaging, and 81% abnormal visual evoked potentials. Our results emphasize the challenges in diagnosing optic neuritis, as not all patients with objectively diagnosed MS exhibit the triad of typical symptoms. To address potentially missing clinical features, incorporating additional paraclinical findings is proposed.

## Introduction

Optic neuritis is an inflammation of the optic nerve that often leads to unilateral visual loss. The diagnosis of optic neuritis is primarily based on the clinical symptoms reported by the patient including reduced visual acuity, eye pain with movements, and deficiency in colour vision (dyschromatopsia)^1–3^. However, optic neuritis can have various underlying causes and heterogenous clinical symptoms, thus objective findings can vary depending on the underlying disease^3–6^. Most commonly, optic neuritis is an initial manifestation of multiple sclerosis (MS) or clinically isolated syndrome suggestive of MS (CIS)^1,7^. A study by the Optic Neuritis Study Group following patients for 15 years after their initial optic neuritis episode demonstrated a risk of developing MS of 50 percent^8^. To diagnose CIS or MS in patients presenting with an optic neuritis as the initial clinical event, the 2017 revisions of the McDonald criteria emphasize the need for objective clinical or paraclinical criteria^7^. Objective findings include a relative afferent pupillary defect (RAPD), abnormal visual evoked potentials (VEP), abnormal magnetic resonance imaging (MRI) findings, abnormal cerebrospinal fluid (CSF) findings, or abnormal optical coherence tomography (OCT) results^7^. It is important to note that the characterization of what is considered clinically typical for optic neuritis and the aforementioned study on the risk of developing MS following optic neuritis have been published more than 10 years ago^2,8^. Since then, there have been various revisions and consensus reports, especially regarding diagnostic criteria for MS^7,9–13^. Since examination methods for patients with reported visual impairment are often not unrestricted available, there is still a risk of a MS misdiagnosis^14,15^. The main challenge lies in the fact that the diagnosis of optic neuritis is primarily made clinically based on subjective statements from patients. In a recent publication, Petzold and colleagues introduced a set of diagnostic guidelines for optic neuritis through a position paper. The aim is to minimize the chances of misidentifying cases of one-sided vision impairment caused by conditions unrelated to optic nerve issues^13^. However, there is currently a lack of a concrete definition on subjective complaints, as well as objective clinical and paraclinical criteria for optic neuritis as the initial manifestation of MS, using the updated 2017 McDonald criteria. The aim of this study was to investigate which combinations of symptoms and objective findings in optic neuritis can indicate the presence of MS in the context of the 2017 McDonald criteria.

## Methods

### Patients

We retrospectively analysed patient records from the Department of Neurology at Hannover Medical School (Hannover, Germany) for the period between 2010 and 2018. This analysis focused on patients admitted with symptoms indicative of optic neuritis. Included were cases where patients reported visual disturbances (such as foggy or blurred vision, visual field defects, reduced visual acuity) in one or both eyes, retrobulbar pain, or dyschromatopsia. As part of the diagnostic process, patients underwent neurological and ophthalmological examinations along with paraclinical tests, including MRI, electrophysiology, and CSF and serum analyses to exclude other potential causes. We excluded patients lacking paired CSF and serum analyses, brain MRI, or ophthalmologic assessment, as well as those presenting with atypical clinical symptoms like double vision, nystagmus, or abrupt monocular vision loss, to ensure the specificity of our analysis towards optic neuritis and related CNS inflammatory conditions. Patients diagnosed with neuromyelitis optica spectrum disorder (NMOSD) according to the 2015 International Panel for NMO Diagnosis (IPND) criteria^16^, or with myelin oligodendrocyte glycoprotein antibody-associated disease (MOGAD)^17^, were grouped into a separate cohort for subgroup analysis.

The 2017 McDonald criteria were retrospectively applied to the patient cohort, resulting in three groups: (1) patients with symptoms of optic neuritis and confirmative objectifiable findings and who also met the criteria for spatial and temporal dissemination and thus were diagnosed with relapsing MS (MS—optic neuritis); (2) patients with symptoms and objective findings of optic neuritis, but without a confirmative diagnosis of MS (CIS—optic neuritis); (3) patients with symptoms suggestive of optic neuritis, but without confirmative objectifiable findings and without assignable disease (suspected optic neuritis—sON). The diagnoses assigned in routine clinical practice for the patients within the sON cohort were “single, isolated optic neuritis” or “idiopathic optic neuritis” in the case of the absence of any pathological abnormalities, and furthermore functional disorders were discussed as the basis for the symptoms stated by the patient. In the case of intrathecal IgG synthesis or an increased CSF cell count as a possible indication of a CNS-specific (autoimmune) inflammatory genesis of the symptoms, the diagnosis of isolated optic neuritis with a suspected (auto)immunological genesis was assigned, most likely corresponding to a “forme fruste” of optic neuritis as defined by Petzold et al.^13^. Definitive final diagnoses with identification of a specific etiological factor for the suspected optic neuritis were, according to the character of the sON cohort, not available. Notably, the diagnosis of CIS was made according to the 2017 McDonald criteria (i.e., objective findings had to be present whereas the presence of brain or spinal cord lesions detected by MRI was not required). Because of the limited number of patients and the challenges in comparing symptoms, individuals with optic neuritis affecting both eyes were not included in the study. All patients who were diagnosed with CIS were investigated for follow-up data. Some of the groups included patients who were previously described. This encompasses a commentary to the optic neuritis criteria recently suggested by Petzold and his team, emphasizing its application to a subset of the patient cohort mentioned^18–20^. This study was approved by the institutional Ethics Committee of the Hannover Medical School (8172_BO_K_2018). Regarding the use of the collected bio-samples, as well as the pseudonymized analysis of the routinely collected parameters, informed consent was obtained from all patients and/or their legal representatives. All investigation and analysis methods were conducted in accordance with the relevant guidelines and regulations.

### Ophthalmologic parameters

All patients underwent ophthalmologic examinations, including visual acuity tests, slit lamp examinations, and assessments for RAPD and dyschromatopsia. Each retrospective analysis of the data was specific to the affected eye. A visual acuity of being able to count fingers without differentiation was considered as 0.01, while the perception of hand movements without differentiation was considered as 0.0052. The exclusive perception of changes in light was equated to a visual acuity of 0^21^. Additionally, the results of visual field perimetry, which were routinely performed, were retrospectively re-evaluated by an ophthalmologist. The visual field deficits were categorized into groups, such as “central scotoma,” “paracentral scotoma,” “complete visual loss,” “no defect,” “unspecific defects,” and “concentric constriction.”

### CSF and serum analysis procedures

CSF and serum analyses were performed at the Neurochemistry Laboratory of the Department of Neurology, Hannover Medical School^19,22^. All included patients with symptoms of optic neuritis were tested for aquaporin 4 (AQP4)- and myelin oligodendrocyte glycoprotein (MOG)- IgG antibodies via established cell-based assays^23,24^. Following microscopic cell counting and differentiation, CSF samples were centrifuged, and total protein levels were measured using the Bradford dye-binding procedure (cut-off = 500 mg/L)^25,26^. Immunoglobulin classes IgG, IgA, IgM, and albumin were quantified in both CSF and serum using kinetic nephelometry (Beckman Coulter IMMAGE). The CSF-serum albumin quotient (QAlb) was used to assess the blood-CSF barrier function. The upper normal reference value for QAlb was calculated using the formula QAlb = 4 + (age in years/15)^26^. Intrathecal synthesis of IgG, IgA, and IgM was determined based on the calculation method outlined by Reiber-Felgenhauer^26^. CSF-specific oligoclonal bands (OCB) were identified through isoelectric focusing in polyacrylamide gels with consecutive silver staining^27^. Quality control measures were regularly conducted for all CSF analytical methods through participation in the external INSTAND survey program^28^.

### Brain MRI

All patients received an MRI of the brain, consisting of at least a T1-weighted, a T2-weighted and a gadolinium-enhanced T1-weighted sequence on MR systems operating at a magnetic field strength of at least 1.5 T. According to the updated McDonald criteria of 2017, the number, location and gadolinium uptake of focal demyelinating lesions were assessed by a board certified neuroradiologist. The image analysis and interpretation of imaging findings were performed according to recent expert panel recommendations^29^. Based on these findings, the dissemination in time and space criteria were applied^7^.

### VEP

VEPs were routinely obtained by trained staff. The pattern reversal rates were at 1.5–2/s, check sizes were 12 mm/41 min. The latency and peak-to-peak amplitude of the initial negative peak (N75) and the subsequent positive peak (P100) were measured. An absolute latency of P100 > 120 ms or a side difference > 10 ms was defined as prolonged VEP^30–32^.

### Application of diagnostic criteria for optic neuritis

The criteria for diagnosing optic neuritis proposed by Petzold and colleagues were applied to our patient cohort^13^. This evaluation was conducted only for patients in the MS and CIS groups, which included individuals with objective signs of optic neuritis. Patients were assigned to the following categories: criteria for possible (1) or definite (2) optic neuritis met, or criteria for optic neuritis not met (3)^13^.

### Statistical analysis

Statistical analysis was performed using GraphPad Prism (version 5.02, La Jolla, CA, USA) and SPSS (version 26, Armonk, NY, USA). The D'Agostino-Pearson normality test was used to assess the normal distribution of values. For non-normally distributed cohorts, the Kruskal–Wallis test with Dunn’s multiple comparisons was applied to compare multiple subgroups. Fisher’s exact test was used for analysing categorical data. Binary logistic regression with a background selection test was utilized to identify the strongest predictors from the baseline characteristics regarding the presence of MS or CIS. Initially all characteristics were included in the model. Thereafter, one element was removed at a time based on its statistical significance until the most significantly predictive one remained. The order in which predictors were removed were determined by their statistical significance. Included were age, sex, as well as the symptoms visual impairment, dyschromatopsia and retrobulbar pain. A significance level of p < 0.05 was considered statistically significant. For the presentation of the results, in non-normally distributed data the median was used together with the 25–75% interquartile range (IQR). Results of the analyses from categorical data, were described with an odds ratio, as well as the corresponding 95% confidence interval (CI).

## Results

### Cohort

**Figure 1:**
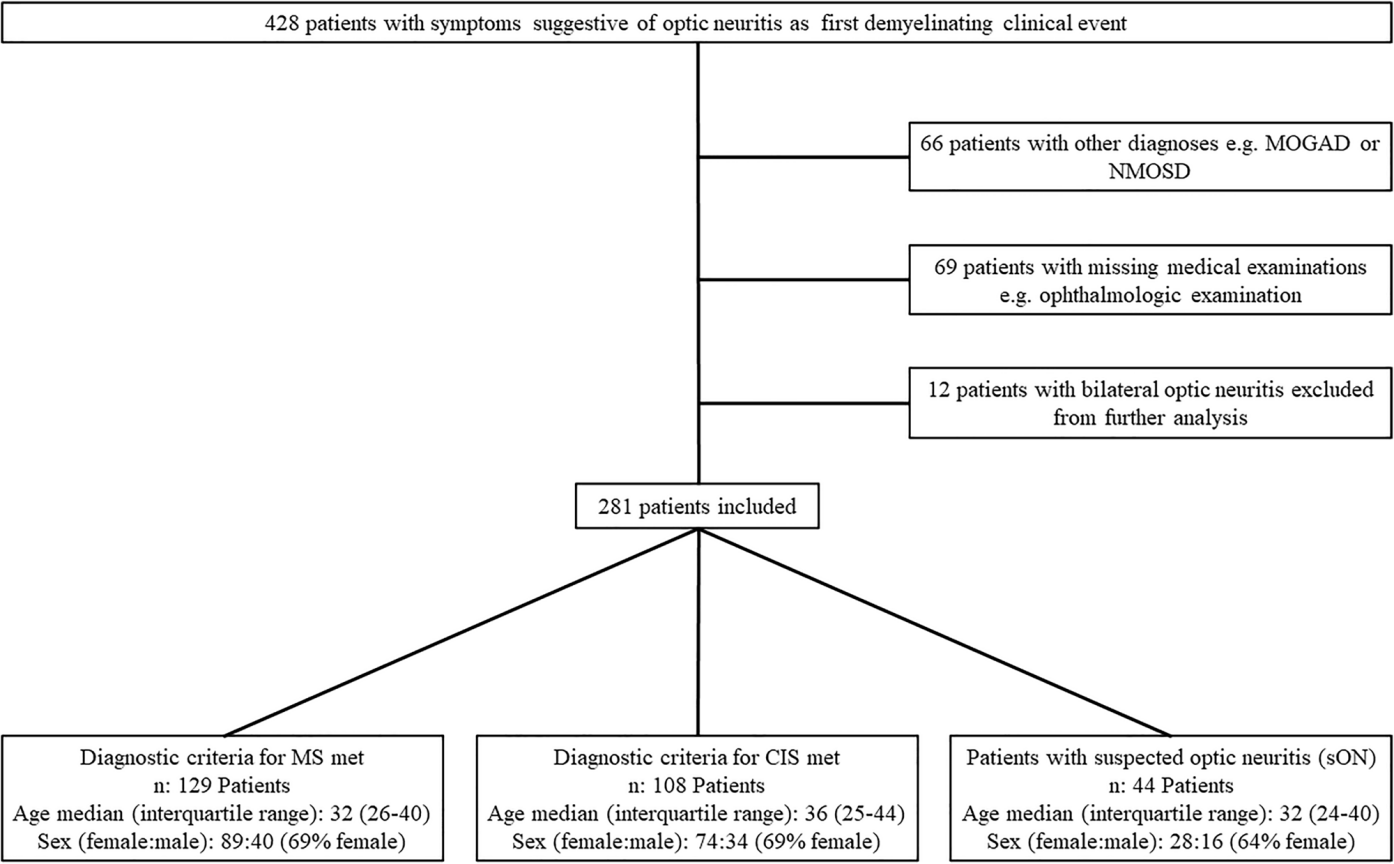
Flow chart of the cohort defined by inclusion and exclusion criteria. CNS, central nervous system; MOGAD, myelin oligodendrocyte glycoprotein antibody-associated disease; NMOSD, neuromyelitis optica spectrum disorder; MS, multiple sclerosis; CIS, clinically isolated syndrome, sON suspected optic neuritis.

Between 2010 and 2018, 428 patients were diagnosed with optic neuritis as their initial clinical event (Fig. 1). Among them, 66 patients received alternative diagnoses such as MOGAD or NMOSD and in 69 patients, medical documentation relevant for further investigation was insufficient. 12 patients with optic neuritis affecting both eyes were excluded. Applying the McDonald criteria 2017 to the remaining 281 patients, 129 were diagnosed with MS, 108 had CIS, and 44 had suspected optic neuritis (sON) without objective findings. The distribution of age and sex was similar across the three groups (Fig. 1). In 39% (42/108) of the CIS patients, a retrospective follow-up could be obtained, with a median follow-up duration of 11.5 months. Of these 42 patients, 43% (18/42) had a change of diagnosis from CIS to MS during the observation period, each time in connection to a relapse event.

### Time between onset of symptoms and hospitalization

The time intervals between symptom onset and presentation at the department of neurology differed among the groups. Only 19% of MS patients and 31% of CIS patients sought medical attention within 1–3 days of symptom onset, while 43% of patients with suspected optic neuritis (sON) did so. Accordingly, the effect reversed within 4–7 days between symptom onset and hospital admission: 42% of MS patients, 27% of CIS patients and 21% of sON patients were admitted to the hospital (Table 1). Comparing the time from symptom onset to hospitalization, there was a significant difference between the MS and the sON group (median MS: 7 days, 25–75% IQR: 4–14; median sON: 4 days, 25–75% IQR: 2–10; p = 0.0162). Overall, there was a trend of later admission from MS to CIS to sON.

**Table 1:** Days between symptom onset and hospital admission.

Group	1–3	4–7	8–14	≥ 15	No data
MS—optic neuritis (n = 129)	19% (25/129)	42% (54/129)	15% (19/129)	19% (24/129)	5% (7/129)
CIS—optic neuritis (n = 108)	31% (34/108)	27% (29/108)	19% (20/108)	20% (22/108)	3% (3/108)
Suspected optic neuritis (n = 44)	43% (19/44)	21% (9/44)	16% (7/44)	9% (4/44)	11% (5/44)

Time measured from patient reported onset of symptoms and time to presentation at hospital in days, groups set for 1–3 days, 4–7 days, 8–14 days and 15 days and longer.

MS, multiple sclerosis; CIS, clinically isolated syndrome; sON, suspected optic neuritis.

### Patient reported symptoms

**Figure 2:**
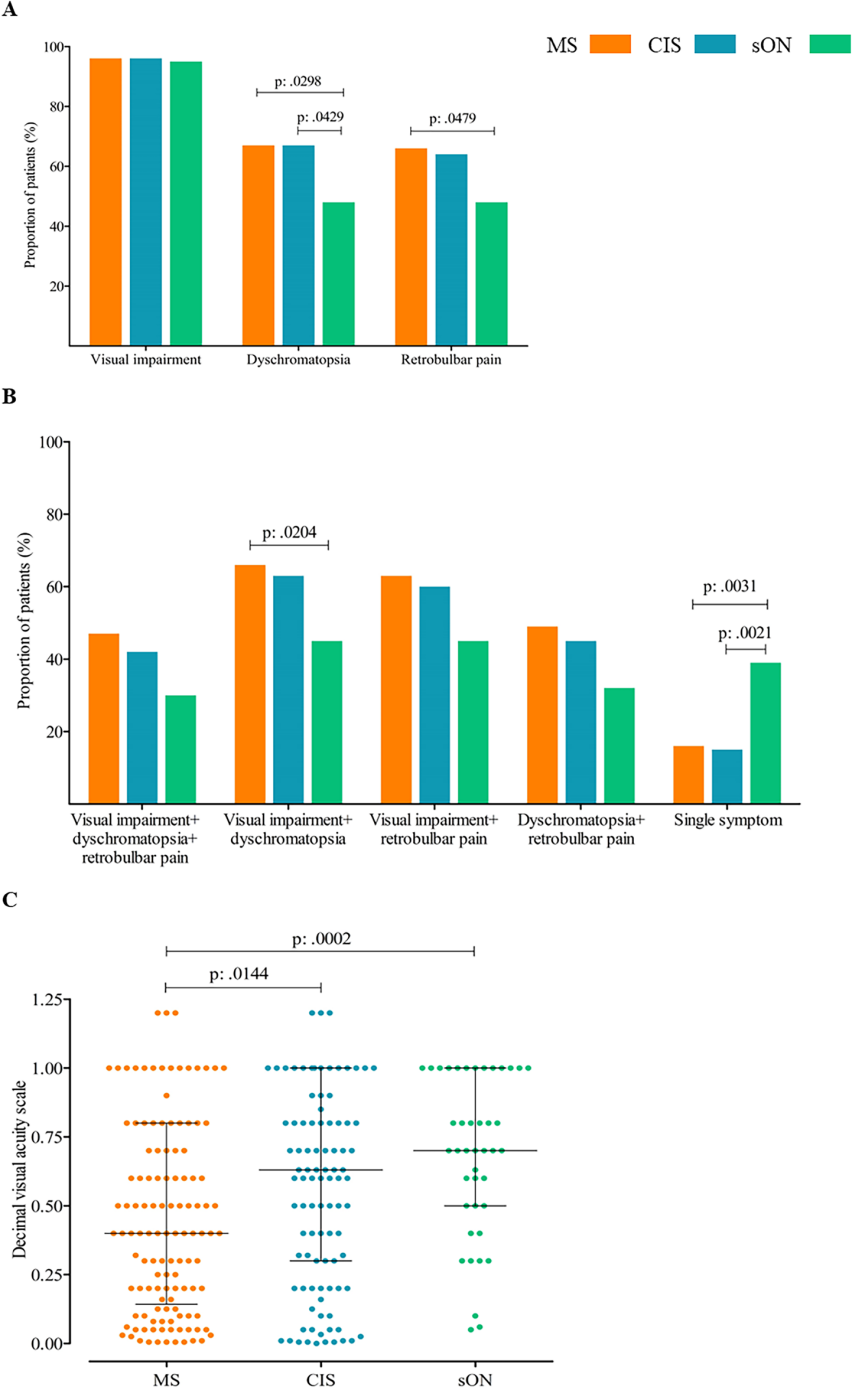
(**A**) Proportion of patients with the respective symptoms at presentation for each group. (MS—optic neuritis, CIS—optic neuritis, suspected optic neuritis). MS, multiple sclerosis; CIS, clinically isolated syndrome. (**B**) Percentage of patients with combination of symptoms at presentation for the respective groups. (MS—optic neuritis, CIS—optic neuritis, suspected optic neuritis). Single symptom, patients with only one of the typical symptoms. MS, multiple sclerosis; CIS, clinically isolated syndrome. (**C**) Visual impairment of patients at initial presentation. Values are given for the different groups: MS—optic neuritis, CIS—optic neuritis, and suspected optic neuritis. Lines and error bars indicate median ± interquartile range. Median values for visual acuity according to the decimal visual acuity scale: MS—optic neuritis: 0.4, CIS—optic neuritis: 0.63, suspected optic neuritis: 0.7. Visual acuity was significantly lower in patients with MS—optic neuritis compared to CIS—optic neuritis (p: 0.0144) and suspected optic neuritis (p: 0.0002). MS, multiple sclerosis; CIS, clinically isolated syndrome; sON, suspected optic neuritis.

The patients' reported symptoms at presentation were categorized into visual impairment, dyschromatopsia, and retrobulbar pain, as well as combinations of these symptoms. Visual impairment was reported by 96% of MS and CIS patients, and 95% of sON patients. Dyschromatopsia was stated by 67% of MS and CIS patients, but only by 48% of sON patients. Retrobulbar pain was reported by 66% of MS patients, 64% of CIS patients, and 48% of sON patients. Dyschromatopsia was significantly more frequent in MS and CIS patients compared to those with sON (MS vs. sON p = 0.0298, OR: 2.27, 95% CI 1.13–4.56; CIS vs. sON p = 0.0429, OR: 2.19, 95% CI 1.07–4.48). Retrobulbar pain was significantly more common in MS patients compared to sON patients (p = 0.0479, OR: 2.12, 95% CI 1.06–4.24) (Fig. 2A).

The combination of all three symptoms was more frequently observed in MS patients (47%), followed by CIS patients (42%), and sON patients (30%). Combinations of two typical optic neuritis-associated symptoms were also more frequent in MS and CIS compared to sON patients (Fig. 2B). The combination of visual impairment and dyschromatopsia was significantly more common in MS patients (p = 0.0204, OR: 2.32, 95% CI 1.16–4.65). Patients with sON were more likely to present with a single symptom compared to MS or CIS patients (sON: 39%, MS: 16%, CIS: 15%; MS vs. sON p = 0.0031, OR: 0.31, 95% CI 0.14–0.66; CIS vs. sON p = 0.0021, OR: 0.28, 95% CI 0.12–0.62). Visual impairment was the most frequently reported single symptom in all three groups. No significant differences were found in the symptom combinations between the MS and CIS groups (Fig. 2B).

Binary logistic regression analysis showed that dyschromatopsia was the strongest marker for the presence of MS compared to sON (p = 0.021, OR: 0.441, 95% CI 0.220–0.885; Table 2). In the comparison between the CIS group and the sON group, dyschromatopsia and age emerged as the strongest markers, with dyschromatopsia having the strongest effect (dyschromatopsia: p = 0.019, OR: 0.417, 95% CI 0.200–0.868; age: p = 0.057, OR: 0.968, 95% CI 0.936–1.001; Table 3).

**Table 2:** Stepwise backward regression of baseline characteristics to test for predictors of the presence of MS vs. sON.

	Variables	p	Odds ratio	95% confidence interval	
				Lower value	Upper value
Step 1^a^	Dyschromatopsia	0.071	0.509	0.244	1.059
	Visual impairment	0.859	0.853	0.148	4.928
	Retrobulbar pain	0.092	0.529	0.252	1.108
	Age at presentation	0.343	0.982	0.947	1.019
	Sex	0.472	0.761	0.362	1.601
	Constant	0.485	2.388		
Step 2^a^	Dyschromatopsia	0.066	0.505	0.244	1.046
	Retrobulbar pain	0.093	0.534	0.256	1.110
	Age at presentation	0.343	0.982	0.947	1.019
	Sex	0.467	0.759	0.361	1.595
	Constant	0.436	2.060		
Step 3^a^	Dyschromatopsia	0.071	0.513	0.248	1.059
	Retrobulbar pain	0.092	0.534	0.257	1.109
	Age at presentation	0.317	0.982	0.946	1.018
	Constant	0.687	1.326		
Step 4^a^	Dyschromatopsia	0.068	0.510	0.248	1.052
	Retrobulbar pain	0.115	0.559	0.272	1.152
	Constant	0.237	0.701		
Step 5^a^	Dyschromatopsia	0.021	0.441	0.220	0.885
	Constant	0.020	0.548		

Results of a binary logistic model with conditional stepwise backward regression to identify the strongest predictors from the baseline characteristics regarding the presence of MS. Included were age, sex, as well as the symptoms visual impairment, dyschromatopsia and retrobulbar pain. MS, multiple sclerosis; sON, suspected optic neuritis.

^a^Variables entered in step 1: dyschromatopsia, visual impairment, retrobulbar pain, age at presentation, sex.

**Table 3:** Stepwise backward regression of baseline characteristics to test for predictors of the presence of CIS vs. sON.

	Variables	p	Odds ratio	95% confidence interval	
				Lower value	Upper value
Step 1^a^	Dyschromatopsia	0.044	0.461	0.217	0.981
	Visual impairment	0.519	0.553	0.091	3.346
	Retrobulbar pain	0.111	0.538	0.251	1.153
	Age at presentation	0.050	0.966	0.933	1.000
	Sex	0.655	0.839	0.390	1.808
	Constant	0.156	6.914		
Step 2^a^	Dyschromatopsia	0.044	0.461	0.217	0.980
	Visual impairment	0.541	0.571	0.095	3.441
	Retrobulbar pain	0.107	0.535	0.250	1.146
	Age at presentation	0.050	0.966	0.933	1.000
	Constant	0.163	5.033		
Step 3^a^	Dyschromatopsia	0.048	0.468	0.221	0.992
	Retrobulbar pain	0.124	0.556	0.263	1.175
	Age at presentation	0.047	0.965	0.933	1.000
	Constant	0.138	2.884		
Step 4^a^	Dyschromatopsia	0.019	0.417	0.200	0.868
	Age at presentation	0.057	0.968	0.936	1.001
	Constant	0.279	2.047		

Results of a binary logistic model with conditional stepwise backward regression to identify the strongest predictors from the baseline characteristics regarding the presence of CIS. Included were age, sex, as well as the symptoms visual impairment, dyschromatopsia and retrobulbar pain. CIS, clinically isolated syndrome; sON, suspected optic neuritis.

^a^Variables entered in step 1: dyschromatopsia, visual impairment, retrobulbar pain, age at presentation, sex.

### Visual impairment

In the MS group, 80% of patients had visual acuity less than 1.0 in the affected eye, compared to 72% in the CIS group, and 66% in the sON group. Evaluation of visual acuity of the affected eye among all patients in each group revealed significant worse visual impairment in MS (median decimal visual acuity: 0.4, 25–75% IQR: 0.14–0.8) patients with optic neuritis compared to CIS (median; 0.63, 25–75% IQR: 0.3–1) and suspected optic neuritis (median; 0.7, 25–75% IQR: 0.5–1) (p = 0.0006). There was no significant difference regarding the visual impairment between CIS and sON patients (Fig. 2C).

### Visual field restrictions

**Figure 3:**
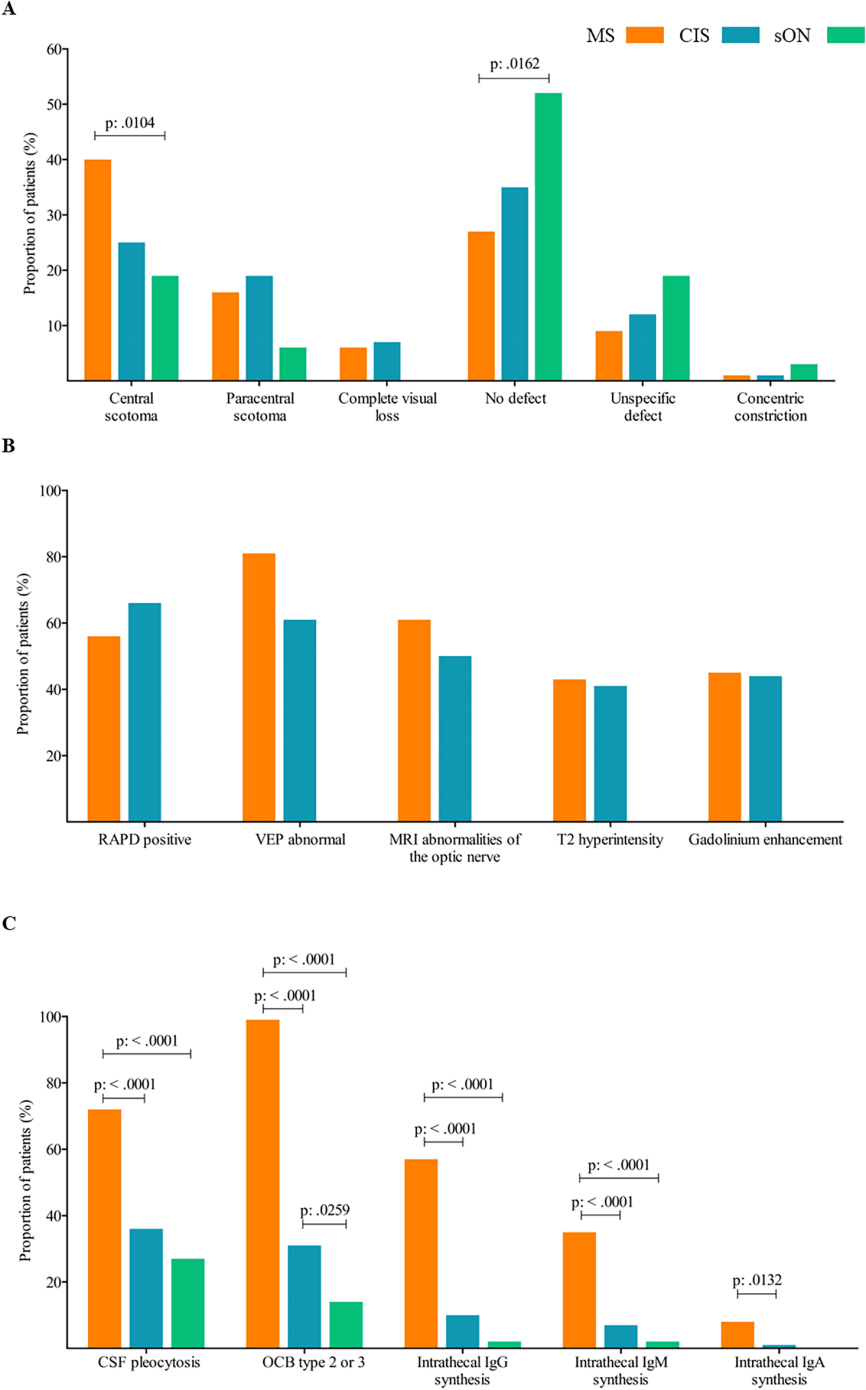
(**A**) Percentage of patients with the respective perimetry abnormalities for each group. (MS—optic neuritis, CIS—optic neuritis, suspected optic neuritis). MS, multiple sclerosis; CIS, clinically isolated syndrome; sON, suspected optic neuritis. (**B**) Proportion of patients with objectifiable findings such as RAPD, abnormalities in MRI of the optic nerve or VEP for the groups MS—optic neuritis and CIS—optic neuritis. By definition, the sON group did not show any objectifiable abnormalities for optic neuritis, as this would at least have meant classification in the CIS group. RAPD, relative afferent pupillary deficit, VEP, visual evoked potential; MRI, magnetic resonance imaging. MS, multiple sclerosis; CIS, clinically isolated syndrome. (**C**) Percentage of patients with the respective CSF finding for each group. (MS—optic neuritis, CIS—optic neuritis, suspected optic neuritis). CSF, cerebrospinal fluid; OCB, oligoclonal bands; MS, multiple sclerosis; CIS, clinically isolated syndrome; sON, suspected optic neuritis.

A subgroup of 198 patients underwent static perimetry, including 99 MS patients, 68 CIS patients, and 31 patients with sON. The percentages of visual field defects refer to this subgroup. Central scotoma was the most common specific visual field defect, occurring in 40% of MS patients, and 25% of CIS patients (Fig. 3A). However, it was significantly less frequent in patients with sON (19%) compared to patients with MS-associated optic neuritis (p = 0.0104, OR: 3.54, 95% CI 1.33–9.42). Paracentral scotoma showed a similar trend towards lower frequency in sON patients. Complete visual loss occurred in 6% of MS patients, and 7% of CIS patients, but none in the sON group. No detectable visual field defect was found in 27% of MS patients, and 35% of CIS patients, with a significantly higher proportion (52%) in the sON group (p = 0.0162, OR: 2.84, 95% CI 1.24–6.54). Non-specific defects were reported by 9% of MS patients, 12% of CIS patients, and 19% of sON patients. Concentric visual field defects were rare, with only one patient in each group exhibiting this finding. Significant differences were observed in the frequency distribution of specific perimetry findings between the MS and sON groups, particularly for “no defect” and “paracentral defect” versus “complete defect” and “central scotoma.” Other groups and perimetry findings showed no significant differences (Table 4).

**Table 4:** Relative frequencies of perimetric defects as possible predictors of MS/CIS vs. sON.

Perimetry; p (OR, 95% confidence interval lower value; upper value)	MS/sON	CIS/sON	MS/CIS
Any defect vs. no defect	0.0162 (2.84, 1.24; 6.54)	0.1847 (1.96, 0.82; 4.63)	0.3066 (1.46, 0.75; 2.83)
Central defect/complete defect vs. paracentral/no defect	0.0104 (3.54, 1.33; 9.42)	0.2328 (1.96, 0.70; 5.48)	0.0784 (1.81, 0.95; 3.45)
Complete loss of vision vs. incomplete loss of vision	0.3347 (4.38, 0.24; 80.02)	0.3212 (5.46, 0.29; 101.9)	0.7595 (0.81, 0.2; 2.78)

Results of a cross-table analysis of perimetry findings at first presentation. Categories were formed as stated in the columns. Fisher’s test was used to test for significant association between the categorical variables. MS, multiple sclerosis; CIS, clinically isolated syndrome; sON, suspected optic neuritis; OR, odds ratio.

### RAPD

RAPD was observed in 56% of patients with optic neuritis and an initial diagnosis of MS. In the CIS group, 66% of patients exhibited a RAPD. By definition, the sON group did not show any objectifiable abnormality with regard to the optic nerve, as this would have meant allocation at least to the CIS group (Fig. 3B).

### Brain MRI

In patients of the MS group, 61% showed optic nerve hyperintensity on T2-weighted images or gadolinium enhancement (43% had T2 hyperintensity and 45% showed gadolinium enhancement). In the CIS group, 50% had pathological findings on MRI (41% with T2 hyperintensity and 44% with gadolinium enhancement) (Fig. 3B).

All MS patients fulfilled the criteria for dissemination in space. In the CIS group, 19% had detectable lesions without meeting the dissemination in space criteria. One patient in the sON cohort had cranial lesions consistent for a possible chronic inflammatory CNS disease, but unrelated to optic nerve or visual pathway. Contrast-enhancing lesions were present in 35% of MS patients and 1% of CIS patients, meeting the MRI criteria for temporal dissemination.

### VEP

Pathologically prolonged VEPs (> 120 ms) were observed in 81% of MS patients and 61% in the CIS group (Fig. 3B).

### Cerebrospinal fluid (CSF)

An increased CSF cell count (> 4 cells/µl) was significantly more frequent in MS patients with optic neuritis (72%) compared to CIS (36%), and sON (27%) patients (MS vs. sON: p =  < 0.0001, OR: 5.28, 95% CI 2.49–11.18; MS vs. CIS: p =  < 0.0001, OR: 3.5, 95% CI 2.07–5.93) (Fig. 3C). The MS group also had a significantly higher CSF cell count compared to the other two groups (p =  < 0.0001; median MS: 7 cells/µl; median CIS: 3 cells/µl; median sON: 1 cell/µl). The CIS and sON groups did not differ significantly.

Regarding intrathecal IgG synthesis according to the Reiber diagram, MS patients (57%) had a higher frequency compared to CIS (10%), and sON (2%) patients (MS vs. sON: p =  < 0.0001, OR: 57.85, 95% CI 7.72–433.3; MS vs. CIS: p =  < 0.0001, OR: 11.86, 95% CI 5.81–24.25) (Fig. 3C). Intrathecal IgM and IgA synthesis were also more frequent in the MS group (IgM 35%, IgA 8%) compared to the CIS group (IgM 7%, IgA 1%) (IgM p =  < 0.0001, OR: 6.7, 95% CI 2.99–15; IgA p = 0.0132, OR: 8.99, 95% CI 1.13–71.45). MS and sON showed a significant difference only in IgM synthesis (sON: IgM synthesis in 2% of patients, p =  < 0.0001, OR: 23.04, CI 3.07–172.9). The CIS and sON groups did not differ significantly for any Ig class.

CSF-specific oligoclonal bands (OCB) showed significantly more positive findings in the MS group (99%) compared to CIS (31%), and sON (14%) (MS vs. sON: p =  < 0.0001, OR: 810.7, 95% CI 94.59–6947; MS vs. CIS: p =  < 0.0001, OR: 278.6, 95% CI 37.34–2078). There was also a significant difference between CIS and sON (CIS vs. sON: p = 0.0259, OR: 2.91, 95% CI 1.12–7.54) (Fig. 3C).

### Application of the 2022 diagnostic criteria for optic neuritis

**Figure 4:**
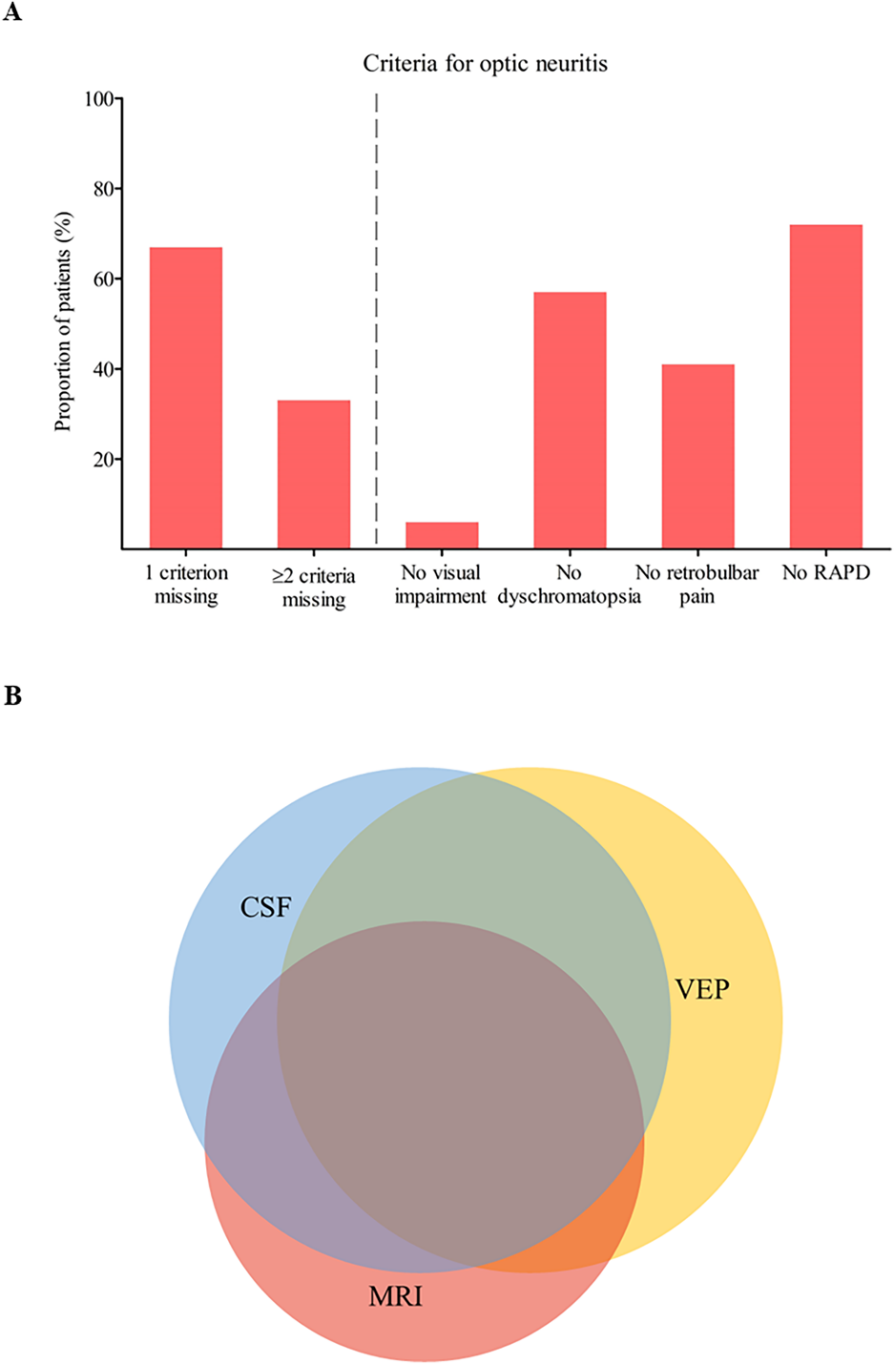
(**A**) Proportion of patients that did not meet diagnostic criteria of optic neuritis with respective missing clinical criteria and available pathological paraclinical findings. RAPD, relative afferent pupillary deficit. (**B**) Venn diagram with pathological paraclinical findings and overlaps of the respective abnormal diagnostics. Abnormal MRI: 43%, CSF abnormalities: 57%, abnormal VEP: 58%, combination of abnormal MRI and CSF: 41%, combination of abnormal MRI and VEP: 36% Combination of abnormal CSF and VEP: 50%, combination of abnormal findings in MRI, CSF and VEP: 29%. MRI, magnetic resonance imaging; CSF, cerebrospinal fluid; VEP, visual evoked potential.

Applying the proposed diagnostic criteria for optic neuritis by Petzold et al.^13^ resulted in the formation of three groups described previously. A subgroup of 203 patients with MS or CIS was formed, with only 41% meeting the criteria for optic neuritis by Petzold and colleagues. Among these patients, 53% met the criteria for possible optic neuritis and 47% for definite optic neuritis. The remaining 59% of patients with MS or CIS did not fulfil the criteria for optic neuritis, primarily due to missing clinical features. Among these patients, 67% lacked one clinical criterion, while 33% lacked two or more criteria. Specific clinical features were absent in these patients, including visual impairment (6%), dyschromatopsia (57%), retrobulbar pain (41%), and RAPD (72%) (Fig. 4A). On the other hand, in terms of objective paraclinical criteria, 43% of patients without clinical criteria for optic neuritis had abnormal MRI findings of the optic nerve, 57% had CSF abnormalities, and 58% had pathological VEP findings. Notably, many patients exhibited abnormalities in multiple paraclinical investigations, with the majority showing pathologies in two or three examinations (Fig. 4B).

### Frequency distribution of clinical characteristics in MOGAD and NMOSD patients

In an additional analysis, the data of patients who presented to the same clinical centre with a suspected diagnosis of optic neuritis and who were diagnosed with NMOSD or MOGAD during diagnostic work-up were retrospectively analysed. A cohort of 25 patients with an initial diagnosis of NMOSD or MOGAD on the basis of unilateral optic neuritis was obtained, 68% were female, the median age was 42 years (IQR 25–75%: 29.5–55). With regard to clinical symptoms, 96% presented with visual impairment, 56% with retrobulbar pain and 52% with dyschromatopsia. The triad of all three symptoms was present in 32%. 43% of patients had RAPD, in two cases, there was no sufficient data available on the RAPD status (Supplementary Table S1).

## Discussion

Diagnosing optic neuritis remains challenging as it heavily relies on patient-reported symptoms, which can vary based on the underlying condition. Distinguishing between isolated optic neuritis and its association with MS is complex, given their similar clinical presentations. Moreover, symptoms like blurred vision and eye pain can imitate other ocular disorders, potentially leading to incorrect diagnoses. Integrating additional diagnostic tests becomes imperative to secure objective and dependable findings that support the optic neuritis diagnosis and enhance diagnostic precision. However, comprehensive data regarding the clinical and paraclinical characteristics of MS-related optic neuritis, the most common cause of optic neuritis, are still lacking^7,8,33^.

In our study, we conducted a thorough analysis of medical records belonging to 281 patients diagnosed with optic neuritis. Our primary aim was to assess the clinical and paraclinical characteristics, particularly concerning their association with MS and CIS. Moreover, we compared this dataset with cases involving isolated optic neuritis. Patients with MS or CIS showed a higher frequency of dyschromatopsia and retrobulbar pain compared to those with suspected optic neuritis. Dyschromatopsia was a distinguishing factor between MS and suspected optic neuritis. Furthermore, patients with MS or CIS often presented with a combination of symptoms, such as dyschromatopsia and retrobulbar pain. These findings suggest that the presence of multiple symptoms may indicate MS as underlying disease. Visual impairment was more pronounced in MS-associated optic neuritis, with central scotoma or complete visual loss being more common. In summary, patients with CIS- and MS-associated optic neuritis exhibited more severe clinical manifestations compared to those without objective findings of optic neuritis.

However, strikingly, only half of patients in whom the diagnosis of optic neuritis led to the diagnosis of MS reported the combination of the typical symptom triad of visual impairment, dyschromatopsia and retrobulbar pain. Using additional objectifiable tests, we found that 56% of patients with MS had RAPD, 61% had MRI abnormalities of the optic nerve, and 81% had abnormal VEP of the affected eye. Although not exclusive to the clinical presentation of optic neuritis, abnormal CSF results were notably more prevalent among individuals with optic neuritis associated with MS in comparison to the other groups. The most commonly observed abnormality was positive oligoclonal bands, which were present in 99% of patients with MS. Remarkably, despite being significantly less frequent than in the other groups, pleocytosis in the CSF was still observed relatively commonly within the sON group, with a cell count exceeding 4/µl in 27% of these patients. This observation emphasizes that in patients in which optic neuritis is suspected after accurate application of typical clinical features, neuroinflammation may be the underlying cause of symptoms even if no objective findings for optic neuritis or an underlying neuroimmunologic disease such as MS, NMOSD or MOGAD can be identified. Our findings support the importance of specific and reliable clinical and paraclinical parameters for the diagnosis of optic neuritis.

The recently proposed diagnostic criteria for optic neuritis suggested further validation in clinical cohorts^13^. In our cohort, a significant portion of patients with MS and optic neuritis did not meet the new diagnostic criteria. The lack of fulfilment of the proposed diagnostic criteria for optic neuritis was primarily due to missing clinical features, which could not be substituted by pathological findings in paraclinical examinations. It is noteworthy that a significant number of these patients had abnormal findings in MRI, CSF, or VEP^20^.

In conclusion, our findings highlight the importance of supplementary paraclinical examinations in facilitating the diagnosis of optic neuritis, particularly in patients with MS. Even though the clinical characteristics stand apart and are more evident in patients with MS, it’s important to take into consideration supplementary objective paraclinical results, like the abnormal prolongation of VEP. Moreover, we suggest that lacking clinical features could potentially be substituted by one or two abnormal paraclinical tests, ensuring a precise optic neuritis diagnosis.

## Limitations of the study

One limitation of this study is the retrospective nature of the analysis. This means that some of the data were incomplete, thus representing a potential bias in the analysis of the data. This, as well as a possible selection bias due to the nature of retrospective patient selection, could be one reason for the high positivity rate of oligoclonal bands in our MS cohort compared to other centres. Furthermore, our data set originates from the clinical environment of a university hospital, which could lead to an additional bias in patient selection. Therefore, a prospective standardized multicentre study should be conducted to validate the data.

### Supplementary Information

Supplementary Table S1.

## Data Availability

The datasets used and analyzed during the current study are available from the corresponding author on reasonable request.
