# Supplementary material for: Clinical and paraclinical characteristics of optic neuritis in the context of the McDonald criteria 2017
Source: Sci Rep. 2024 Mar 27;14:7293. doi: 10.1038/s41598-024-57199-4 (PMC10973511; doi:10.1038/s41598-024-57199-4)
Supplement: Supplementary file 1 — Supplementary Table S1. [file 41598_2024_57199_MOESM1_ESM.pdf]

Clinical and paraclinical characteristics of optic neuritis in the context of the McDonald criteria 2017

K. F. Jendretzky[1], A. Bajor[2], LM. Lezius[1], M. W. Hümmert[1], FF. Konen[1], GM. Große[1], P. Schwenkenbecher[1], KW. Sühs[1], C. Trebst[1], C. Framme[2], M. P. Wattjes[3], S. G. Meuth[4], S. Gingele[1*], T. Skripuletz[1*#]

Supplementary Table S1: Frequency distribution of clinical characteristics in MOGAD and NMOSD patients

	**All**	**MOGAD**	**NMOSD**
**Number of patients**	25	14	11
**Female, % (n)**	68% (17/25)	71% (10/14)	64% (7/11)
**Age, median (25%-75% IQR)**	42 (29.5-55)	42.5 (28.5-51)	42 (36-61)
**Visual phenomena, % (n)**	96% (24/25)	93% (13/14)	100% (11/11)
**Retrobulbar pain, % (n)**	56% (14/25)	64% (9/14)	45% (5/11)
**Dyschromatopsia, % (n)**	52% (13/25)	50% (7/14)	55% (6/11)
**Combination of all three, % (n)**	32% (8/25)	29% (4/14)	36% (4/11)
**RAPD positive, % (n)**	43% (10/23)	33% (4/12)	55% (6/11)

Analysis of the frequency distribution of clinical characteristics in MOGAD and NMOSD patients. MOGAD, Myelin oligodendrocyte glycoprotein antibody-associated disease; NMOSD, neuromyelitis optica spectrum disorder; RAPD, relative afferent pupillary defect.
